# Predictors of loss to follow-up before HIV treatment initiation in Northwest Ethiopia: a case control study

**DOI:** 10.1186/1471-2458-13-867

**Published:** 2013-09-22

**Authors:** Ismael Ahmed, Salem T Gugsa, Seblewengel Lemma, Meaza Demissie

**Affiliations:** 1 University of Gondar and Addis Continental Institute of Public Health, P.O. Box 180432, Addis Ababa, Ethiopia; 2 Department of Global Health, University of Washington, 901 Boren Avenue, Suite 1100, Seattle, Washington 98104, USA; 3 Addis Continental Institute of Public Health, P.O. Box 26751/1000, Addis Ababa, Ethiopia

**Keywords:** Pre-antiretroviral treatment loss to follow-up, HIV patients, Case control, Ethiopia, Africa

## Abstract

**Background:**

In Ethiopia, there is a growing concern about the increasing rates of loss to follow-up (LTFU) in HIV programs among people waiting to start HIV treatment. Unlike other African countries, there is little information about the factors associated with LTFU among pre-antiretroviral treatment (pre-ART) patients in Ethiopia. We conducted a case–control study to investigate factors associated with pre-ART LTFU in Ethiopia.

**Methods:**

Charts of HIV patients newly enrolled in HIV care at Gondar University Hospital (GUH) between September 11, 2008 and May 8, 2011 were reviewed. Patients who were “loss to follow-up” during the pre-ART period were considered to be cases and patients who were “in care” during the pre-ART period were controls. Logistic regression analysis was used to explore factors associated with pre-ART LTFU.

**Results:**

In multivariable analyses, the following factors were found to be independently associated with pre-ART LTFU: male gender [Adjusted Odds Ratio (AOR) = 2.00 (95% CI: 1.15, 3.46)], higher baseline CD4 cell count (251–300 cells/μl [AOR = 2.64 (95% CI: 1.05, 6.65)], 301–350 cells/μl [AOR = 5.21 (95% CI: 1.94, 13.99)], and >350 cells/μl [AOR = 12.10 (95% CI: 6.33, 23.12)] compared to CD4 cell count of ≤200 cells/μl) and less advanced disease stage (WHO stage I [AOR = 2.81 (95% CI: 1.15, 6.91)] compared to WHO stage IV). Married patients [AOR = 0.39 (95% CI: 0.19, 0.79)] had reduced odds of being LTFU. In addition, patients whose next visit date was not documented on their medical chart [AOR = 241.39 (95% CI: 119.90, 485.97)] were more likely to be LTFU.

**Conclusion:**

Our study identified various factors associated with pre-ART LTFU. The findings highlight the importance of giving considerable attention to pre-ART patients’ care from the time that they learn of their positive HIV serostatus. The completeness of the medical records, the standard of record keeping and obstacles to retrieving charts also indicate a serious problem that needs due attention from clinicians and data personnel.

## Background

Sub-Saharan Africa bears the lion’s share of the global Human Immunodeficiency Virus (HIV) burden, with 22.5 million infected people, which constitutes 68% of the global total [1]. According to the Ethiopian HIV related estimates and projections for the year 2012, there are759268 People Living with HIV/AIDS (PLHIV) in Ethiopia which is 1.3% of the national adult HIV prevalence. The Amhara region, which is one of the nine regional states of Ethiopia located in the northern part of the country, carries 27% of the national HIV burden with an estimated 1.4% adult HIV prevalence [2]. Ethiopia introduced a fee based Anti-Retroviral Treatment (ART) program in 2003. Since 2005, much progress has been made in making free ART available to a large number of Ethiopians [3]. By the end of June 2011, out of 580909 PLHIV who had been enrolled in chronic care, 333434 (57%) patients had started ART but only 247805 (74%) were currently on treatment [4].

There is a growing concern about the increasing rates of LTFU in HIV programs among people already on treatment and those waiting to start HIV treatment. Studies from Ethiopia, South Africa, Uganda, and Cambodia have demonstrated that there is a high rate of pre-ART mortality and LTFU [5-9]. However, compared to patients who have started ART, less emphasis has been given to the follow-up of pre-ART patients, making them a neglected population [5,10,11]. Unlike the follow-up of PLHIV who are taking ART, there is no standardized definition of LTFU and appointment system for pre-ART patients and this contributes to the challenges of tracing those LTFU in Ethiopia [5]. This gap highlights the need for greater focus upon retention of pre-ART patients from the beginning of HIV care, not just after patients have been initiated on ART. In Ethiopia, there is little information about the magnitude of the problem or the associated factors that contribute to LTFU among pre-ART patients. Therefore, this study set out to identify factors associated with pre-ART LTFU among newly enrolled HIV-infected patients.

## Methods

### Study design

The study design was a case–control study using chart review of HIV patients newly enrolled in pre-ART care retrospectively between September 11, 2008 and May 8, 2011.

### Study setting

The study was conducted in Gondar University Hospital, Gondar town in the Amhara region of Ethiopia, about 735 kilometers north of the capital city Addis Ababa. The hospital has 400 beds and serves more than five million people. The hospital began ART service provision in March 2005. In August 2007, the hospital started HIV/AIDS case management program by deploying trained adherence case managers and adherence supporters to assess patients’ needs, develop patient-centered care plan, and provide adherence counseling, health education, psychosocial support, trace patients who are LTFU, monitor patients “at-risk for non-adherence” and link patients to community resources [12]. According to the Ethiopian guideline for the implementation of HIV/AIDS case management, adherence case managers are trained non-health care professionals who are high school graduate with some experience on HIV/AIDS. Adherence supporters are PLHIV with a minimum of an 8^th^ grade education that are enrolled in HIV care and demonstrated good adherence to HIV care. The guideline sets clinical and social criteria including poor adherence, history of other chronic illnesses, history of substance abuse, problems of accessing food, lack of shelter, lack of psychosocial support, etc. to screen and identify “at-risk for non-adherence” patients or patients who may likely not adhere to treatment and care and potentially become LTFU [3].

Patients are linked to the HIV clinic either from HIV testing clinics within the hospital or formally referred (transferred in) by other health facilities. At presentation to the clinics, all patients undergo a complete assessment using standardized intake forms whereby a full clinical history and examination is conducted to determine WHO HIV disease staging and screen for the presence of opportunistic diseases. According to the national guideline, HIV-positive adults and adolescents are considered eligible for ART if they are in WHO clinical stage IV (irrespective of CD4 count), or in stage III if the CD4 cell count is ≤350 cells/μl, or in any of the WHO clinical stages if the CD4 cell count is <200 cells/μl. ART is also recommended for all patients with active pulmonary tuberculosis (TB) with CD4 cell count <350 cells/μl, or patients with extra-pulmonary or disseminated TB irrespective of their CD4 cell count. If CD4 cell count measurement is not available, all patients with active TB are eligible for ART. Non-eligible patients are provided with an appointment to attend the hospital every three months for cotrimoxazole preventive therapy (CPT) and every six months for laboratory evaluation (including measurement of CD4 cell count) [13].

In GUH, patients who are eligible for ART receive preliminary counseling and then are asked to return home with a cotrimoxazole prescription (if applicable) and prepare for ART initiation within two weeks. They are requested to come back to the hospital accompanied by a relative, if possible, to foster a supportive home environment and reinforce the importance of treatment adherence. All new HIV positive patients who are enrolled into chronic HIV care are linked to adherence case managers for counseling and education on HIV infection and the importance of adherence to care. The adherence case managers also conduct a needs assessment to identify patients who are “at-risk for non-adherence” [3]. There is a tracking system in place to trace patients who are LTFU during the pre-ART and ART periods. However, unlike ART patients who are labeled as “lost” when patients have not seen for more than one month and “dropped” when having been loss to follow-up for over three months, there is no standardized definition of LTFU being used for pre-ART patients. This makes it a challenge to track pre-ART patients and identify LTFU in a timely fashion [5]. By the end of June 2011, a total of 9236 HIV patients had been enrolled in HIV chronic care service and 6178 had been initiated on ART. At the time of the study, the ART clinic was equipped with a total of 27 trained staff, including one physician, one health officer, eight nurses, two pharmacy technicians, three adherence case managers, eight adherence supporters, and four data personnel.

### Study participants

The study population was those HIV positive patients who were newly enrolled and followed up in the pre-ART care at GUH from September 11, 2008 to May 8, 2011. HIV patients who were aged <15 years old, pregnant, transferred in from another health facility, transferred out to another health facility and known to have died before starting ART were excluded from the study. In addition to this, those patients who started ART within the first two weeks following pre-ART enrollment were excluded from the study due to the short pre-ART follow-up period.

### Variables

In this study, the explanatory variables were socio-demographic and clinical variables including age, gender, marital status, level of education, religion, residence, patient referral information, employment status, HIV disclosure status, history of TB, baseline CD4 count, and WHO staging and the dependent variable was pre-ART LTFU. The major pre-ART outcome was defined and analyzed as 'pre-ART loss to follow-up’ or 'in care’.

### Definitions

We defined 'Pre-ART loss to follow-up’ (cases) to include patients who were not on ART and did not return for care for a period of one month or more since their most recent documented appointment date, [5,14,15]. For patients whose next appointment date was not documented, 'pre-ART loss to follow-up’ included patients who were not on ART and did not return to care for seven months or more since their last hospital visit.

Patients who were 'in care’ (controls) were defined as patients who were alive, followed pre-ART care as scheduled [16], visited the hospital within seven months of their last visit, or were currently on ART.

### Measurement

The data sources were the Federal Ministry of Health patients’ registration database and intake forms which are completed during the enrollment of HIV patients in chronic care. The data abstraction tool for chart review was prepared based on the information contained within the patient registration and follow-up card, including the socio-demographic and clinical variables of interest. Once eligible patients for the study were identified based on the inclusion criteria, study data was abstracted by four experienced ART prescriber nurses.

Patients’ length of follow-up time in pre-ART care was grouped in to less than one month, one to six months, seven to 12 months and greaterthan12 months for analysis. Accordingly, LTFU patients who only had the first visit to HIV clinic were categorized under less than one month follow-up time in pre-ART care.

### Study size

The sample size was calculated using the following assumptions for an unmatched case–control study design: 95% confidence interval (CI), 80% power, 25% level of exposure in the 'in care’ (control) group, 1:2 ratio of cases-to-controls, and 1.5 expected odds ratio. According to the data from GUH HIV clinic, about 3000 PLHIV were enrolled in pre-ART care in the period of September 11, 2008 to May 8, 2011, amounting to 32 months of cohort data to be studied. After identifying the list of all eligible study subjects, cases and controls were listed separately and simple random sampling technique was used to select the sample cases and controls using SPSS version 18.

### Data analysis

Data coding, entry and cleaning was carried out using Epi Info version 3.5.1 and the analysis was carried out using SPSS version 18. Univariate analysis was carried out to describe baseline socio-demographic and clinical characteristics of the study subjects using simple frequency distribution. Chi-squared tests were used to measure the significance of differences in socio-demographic and clinical characteristic between cases and controls. Confounders were controlled for during the analysis stage using logistic regression and computed adjusted odds ratios with 95% confidence intervals. We have also checked for interaction between variables using the logistic regression after creating the interaction terms.

Charts with incomplete data were entered as “missing” and the rate of missing data for different variables was below 5% except the employment status variable which was excluded without replacement from the logistic regression analysis.

### Ethical approval

Ethical clearance was obtained from the institutional review board of the University of Gondar and Addis Continental Institute of Public Health. Confidentiality of patients’ data was ensured. While reviewing patients’ records, non-personal identifiers such as patients’ medical registration number and pre-ART number were used to distinguish study subjects during data collection. We communicated the list of LTFU patients to adherence supporters for future tracing to establish their current status and assist them to resume their care.

## Results

**Figure 1:**
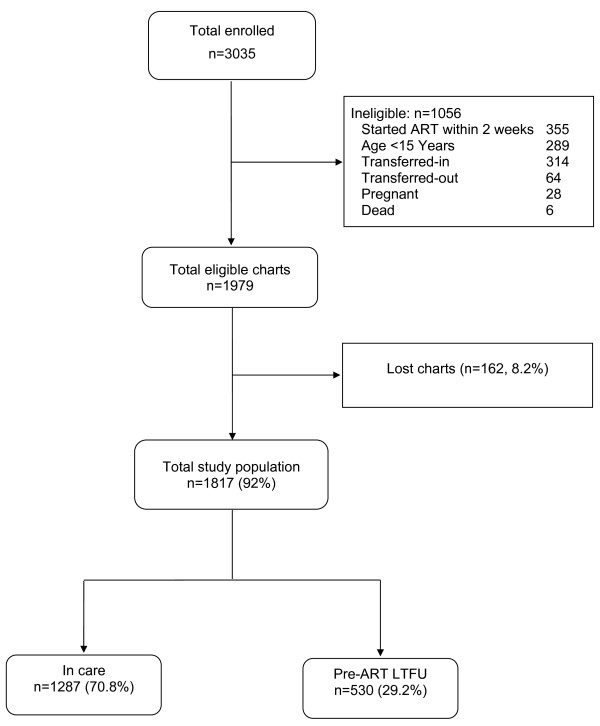
Profiles of HIV clients at GUH (September 11, 2008 to May 8, 2011).

Between September 11, 2008 and May 8, 2011, a total of 3035 (1240 males and 1795 females) HIV patients were newly enrolled for HIV care at GUH ART clinic. Of these, 1979 were eligible for this study based on the inclusion criteria. Of the eligible patients, 8% (n=162) of pre-ART patients were excluded from the study because their charts could not be found. Out of the remaining 1817 study population, 70.8% (n=1287) were patients who were under active follow-up for their HIV care while the other 29.2% (n=530) were lost to follow-up during the pre-ART period (Figure 1). In this study, a total of 1089 (363 cases and 726 controls) patient charts were reviewed from 1817 study population.

### Baseline socio-demographic characteristics of the study population

Out of the 1089 study subjects analyzed, 59.7% (650) were female. The majority (59.5%; n=316) of patients who were pre-ART LTFU were also females. By the time of enrollment into HIV care, nearly half (45.7%; n=498) of the study subjects were between 25–34 years of age. The mean age distribution was 32.7 years with a standard deviation of 9.12. Of the total study population, 45.7% (n=498) were married. With regards to educational status of study subjects, 38.3% (n=417) of patients completed secondary school or above followed by 35.3% (n=384) of patients with no education. The majority (91%; n=991) of study subjects were followers of orthodox Christianity. Just over one quarter (26.9%; n=293) of the study subjects enrolled in HIV care lived outside the city of Gondar (Table 1).

**Table 1:** Baseline socio-demographic characteristics of pre-ART patients at GUH (September 11, 2008 to May 8, 2011)

**Variables**	**Total, n (%)**	**In care, n (%)**	**LTFU, n (%)**	**P-value**
Gender (N=1089)				0.930
Male	439 (40.3)	292 (40.2)	147 (40.5)	
Female	650 (59.7)	434 (59.8)	216 (59.5)	
Age group (N=1089)				<0.001
15-24	176 (16.2)	93 (12.8)	83 (22.9)	
25-34	498 (45.7)	327 (45.0)	171 (47.1)	
35-44	283 (26.0)	207 (28.5)	76 (20.9)	
44+	132 (12.1)	99 (13.6)	33 (9.1)	
Marital status (N=1089)				0.013
Never married	190 (17.4)	113 (15.6)	77 (21.2)	
Married	498 (45.7)	351 (48.3)	147 (40.5)	
Divorced/separated	319 (29.3)	202 (27.8)	117 (32.2)	
Widow/er	82 (7.5)	60 (8.3)	22 (6.1)	
Educational status (N=1089)				0.010
No education	384 (35.3)	244 (33.6)	140 (38.6)	
Primary	288 (26.4)	181 (24.9)	107 (29.5)	
Secondary & above	417 (38.3)	301 (41.5)	116 (32.0)	
Religion (N=1089)				0.131
Muslim	87 (8.0)	55 (7.6)	32 (8.8)	
Orthodox	991 (91.0)	661 (91.0)	330 (90.9)	
Others	11 (1.0)	10 (1.4)	1 (.3)	
Place of residence (N=1089)				0.411
Gondar	796 (73.1)	525 (72.3)	271 (74.7)	
Out of Gondar	293 (26.9)	201 (27.7)	92 (25.3)	
Employment status (N= 995)				0.186
Employed	568 (57.1)	382 (58.6)	186 (54.2)	
Unemployed	427 (42.9)	270 (41.4)	157 (45.8)	
Availability of emergency contact person (N=1089)				0.396
Yes	955 (87.7)	641 (88.3)	314 (86.5)	
No	134 (12.3)	85 (11.7)	49 (13.5)	

When we compared cases with controls, the age distribution (p≤0.001), marital status (p=0.013) and educational status (p=0.010) were significantly different in cases and controls. A higher proportion of LTFU individuals (22.9%) were younger (age 15–24) when compared to those who were in care (12.8%). Higher proportions of LTFU individuals had never been married (21.2%) or were divorced or separated (32.2%) compared to those who were in care (15.6% and 27.8%, respectively). Moreover, a higher proportion of LTFU individuals had less education (no education, 38.6%; primary level education, 29.5%) compared to those who were in care (33.6% and 24.9%, respectively) (Table 1).

### Baseline clinical characteristics and chronic care follow-up status

Among the 1076 patients who had their baseline CD4 cell count documented, 56.8% (n=611) had CD4 count ≤200 cell/μl and 20.0% (n=215) had a CD4 count >350 cell/μl. In contrast, among the 1070 charts that had complete information about WHO staging, the majority (41.7%; n=446) of patients were stage III followed by 24% (n=257) stage I patients. Among 1044 patient charts that had documentation on past history of tuberculosis, 21.3% (n=222) patients had a history of tuberculosis treatment.

The distribution of CD4 cell count categories, WHO stage and recorded next appointment date were significantly different in cases versus controls (p≤0.001). A higher proportion of LTFU patients (42.2%) had a CD4 count >350 cell/μl when compared to those who were in care (9.1%). Similarly, higher proportions of LTFU patients (34%) were in WHO stage I compared to those who were in care (19.1%). Individuals who were in care (97.8%) were more likely to have their next appointment date recorded than those that were LTFU (20.4%) (Table 2).

**Table 2:** Baseline clinical characteristics of pre-ART patients at GUH (September 11, 2008 to May 8, 2011)

**Variables**	**Total, n (%)**	**In care, n (%)**	**LTFU, n (%)**	**P-value**
Patient referral information (N= 1081)				0.118
Within the hospital	796 (73.6)	518 (72.1)	278 (76.6)	
Outside the hospital	285 (26.4)	200 (27.9)	85 (23.4)	
Disclosure of HIV+ status (N=1089)				0.174
To spouse/family members only	616 (56.6)	423 (58.3)	193 (53.2)	
To relatives/friends only	86 (7.9)	61 (8.4)	25 (6.9)	
To spouse/family members & relatives/friends	35 (3.2)	23 (3.2)	12 (3.3)	
To no one	352 (32.3)	219 (30.2)	133 (36.6)	
CD4 cell count/μl (N= 1076)				<0.001
≤ 200	611 (56.8)	505 (69.8)	106 (30.0)	
201-250	111 (10.3)	73 (10.1)	38 (10.8)	
251-300	75 (7.0)	50 (6.9)	25 (7.1)	
301-350	64 (5.9)	29 (4.0)	35 (9.9)	
> 350	215 (20.0)	66 (9.1)	149 (42.2)	
WHO stage (N= 1070)				<0.001
Stage I	257 (24.0)	137 (19.1)	120 (34.0)	
Stage II	194 (18.1)	126 (17.6)	68 (19.3)	
Stage III	446 (41.7)	332 (46.3)	114 (32.3)	
Stage IV	173 (16.2)	122 (17.0)	51 (14.4)	
History of TB treatment (N= 1044)				0.058
Yes	222 (21.3)	159 (23.0)	63 (17.9)	
No	822 (78.7)	533 (77.0)	289 (82.1)	
Next appointment recorded (N= 1089)				<0.001
Yes	784 (72.0)	710 (97.8)	74 (20.4)	
No	305 (28.0)	16 (2.2)	289 (79.6)	

Concerning the length of follow-up time in HIV chronic care, most (61.7%; n=672) of the patients had less than one month follow-up during their pre-ART period. The median length of time in the pre-ART period was less than one month, ranging from less than one up to 37 months. Out of a total of 363 pre-ART LTFU cases, 86% (n=312) had less than one month of follow-up, 10.4% (n=38) had between one and six months of follow-up, and 3.6% (n=13) had more than seven months of follow-up time in the clinic. When we examine the time of LTFU of the 363 pre-ART LTFU patients, 84.8% (n=308) of LTFU patients failed to return since their first clinic visit or did not show up after the date of enrollment.

Regarding the practice of clinicians on documenting the next appointment date of patients in pre-ART care, the next appointment date was not documented on patients’ medical record for more than a quarter (28%; n=305) of patients (Table 2).

### Socio-demographic and clinical factors associated with pre-ART LTFU

After controlling for the effects of gender, age, marital status, educational status, place of residence, HIV disclosure, history of tuberculosis treatment, CD4 cell count, WHO stage, and record of next appointment, five variables were found to be associated significantly with pre-ART LTFU. As shown in Table 3, males had twice the odds of being LTFU during the pre-ART period compared to females (adjusted OR = 2.00 (95% CI: 1.15, 3.46)). Married patients had a 61% reduced odds of being LTFU during the pre-ART period compared to those who had never been married (adjusted OR = 0.39 (95% CI: 0.19, 0.79)). Compared to patients with a baseline CD4 count ≤200 cells/μl, the adjusted odds ratio for being LTFU before starting ART was 2.64 (95% CI: 1.05, 6.65), 5.21 (95% CI: 1.94, 13.99), and 12.10 (95% CI: 6.33, 23.12) for patients with a CD4 count of 251–300 cells/μl, 301–350 cells/μl, and >350 cells/μl respectively. Similarly, patients who were in WHO stage I at baseline, had an adjusted odds ratio for LTFU during the pre-ART period of 2.81 (95% CI: 1.15, 6.91) when compared to patients who were in WHO stage IV.

**Table 3:** Socio-demographic and clinical factors associated with pre-ART loss to follow-up at GUH

		**Crude OR (95% CI)**			**Adjusted**^**+**^**OR (95% CI)**	
**Variables**	**n total**	**n (%)**	**OR**	**95% CI**	**OR**	**95% CI**
Gender	1089					
Male		439 (40.3)	1.01	(0.78, 1.31)	2.00	(1.15, 3.46)*
Female		650 (59.7)	1.00		1.00	
Age group	1089					
15-24		176 (16.2)	1.00		1.00	
25-34		498 (45.7)	0.59	(0.41, 0.83)*	0.80	(0.40, 1.61)
35-44		283 (26.0)	0.41	(0.28, 0.61)*	0.63	(0.28, 1.41)
44+		132 (12.1)	0.37	(0.23, 0.61)*	0.77	(0.30, 2.00)
Marital status	1089					
Never married		190 (17.4)	1.00		1.00	
Married		498 (45.7)	0.62	(0.43, 0.87)*	0.39	(0.19, 0.79)*
Divorced/separated		319 (29.3)	0.85	(0.59, 1.23)	1.18	(0.57, 2.44)
Widow/er		82 (7.5)	0.54	(0.31, 0.95)*	0.54	(0.18 , 1.68)
Educational status	1089					
No education		384 (35.3)	1.00		1.00	
Primary		288 (26.4)	1.03	(0.75, 1.41)	1.47	(0.78, 2.77)
Secondary & above		417 (38.3)	0.67	(0.50, 0.91)*	0.88	(0.48, 1.63)
Disclosure of HIV+ status	1089					
To spouse/family members only		616 (56.6)	1.00		1.00	
To relatives/friends only		86 (7.9)	0.90	(0.55, 1.47)	0.38	(0.12, 1.19)
To spouse/family members & relatives/friends		35 (3.2)	1.14	(0.56, 2.35)	1.47	(0.41, 5.32)
To no one		352 (32.3)	1.33	(1.01, 1.75)*	0.65	(0.37, 1.14)
Place of residence	1089					
Gondar		796 (73.1)	1.00		1.00	
Out of Gondar		293 (26.9)	0.89	(0.67, 1.18)	1.07	(0.61, 1.87)
History of TB treatment	1044					
Yes		222 (21.3)	1.00		1.00	
No		822 (78.7)	1.37	(0.99, 1.89)	0.73	(0.39 , 1.35)
CD4 cell count/μl	1076					
≤ 200		611(56.8)	1.00		1.00	
201-250		111(10.3)	2.48	(1.59, 3.87)*	2.22	(0.95, 5.19)
251-300		75 (7.0)	2.38	(1.41, 4.02)*	2.64	(1.05, 6.65)*
301-350		64 (5.9)	5.75	(3.37, 9.82)*	5.21	(1.94, 13.99)*
> 350		215 (20.0)	10.76	(7.52, 15.38)*	12.10	(6.33, 23.12)*
WHO stage	1070					
Stage I		257 (24.0)	2.10	(1.39, 3.15)*	2.81	(1.15, 6.91)*
Stage II		194 (18.1)	1.29	(0.83, 2.01)	1.75	(0.69, 4.45)
Stage III		446 (41.7)	0.82	(0.56, 1.21)	1.72	(0.77, 3.81)
Stage IV		173 (16.2)	1.00		1.00	
Next appointment record	1089					
Yes		784 (72.0)	1.00		1.00	
No		305 (28.0)	173.30	(99.25, 302.61)*	241.39	(119.90, 485.97)*

*OR*: Odds Ratio, *CI*: Confidence Interval, * p≤0.05, ^+^A total of 1015 subjects included in the adjusted model.

Patients whose next appointment date was not documented on their chart had substantially increased odds of being LTFU during the pre-ART period compared to those who had a record of the next appointment date documented (adjusted OR = 241.39 (95% CI: 119.90, 485.97).

## Discussion

This study is one of the few reports from a resource limited-setting to describe the factors associated with LTFU among a pre-ART population of PLHIV. This group of patients has not been well studied compared to PLHIV who are receiving ART. This is despite emerging evidence of the poor disease outcomes in this pre-ART group. This highlights the need for greater focus upon retention of pre-ART patients from the beginning of HIV care, and not just after being initiated on ART [5,10,11]. Our study has examined the socio-demographic and clinical factors associated with LTFU during the pre-ART period in Ethiopia and found that male gender, being unmarried, CD4 cell count >250 cells/μl, WHO stage I, and not having documented next appointment date on the medical chart were all associated significantly with increased odds of pre-ART LTFU.

Similar with a study conducted in southern Ethiopia [5], the majority of patients in this study started care after reaching compromised immune system. This was shown by the high proportion of patients that had baseline CD4 count of ≤200 cells/μl and WHO stage III and IV. In this study males were found to be at higher risk of LTFU before starting ART. This finding was consistent with studies conducted in Uganda [17] and Malawi [14]. Unlike the findings of previous studies, this study identified an association between marital status and pre-ART LTFU. In this study being married was associated with lower odds of pre-ART LTFU compared to those who had never married. This could be explained by the importance of family support in HIV care [18,19].

Low CD4 cell count was identified as an important risk factor for pre-ART LTFU at a semi-private hospital in Durban, South Africa [16] and in a non-governmental clinic in Jinja, Uganda [17]. In studies conducted in Malawi and Kenya, having an advanced WHO stage of illness (III and IV) was identified as a risk factor for pre-ART LTFU [14]. Our study findings contradicted this, with increased odds of LTFU among patients with better immune system indicators (higher CD4 cell count and less advanced WHO stage), and this could be due to methodological differences with respect to operational definitions and also differences between the study settings. Unlike our study, which defined pre-ART patients as all newly enrolled HIV patients who did not start ART, studies conducted in South Africa [16], Uganda [17], Malawi and Kenya [14] focused on pre-ART patients with low CD4 cell count and advanced WHO stage. These groups of patient are not representative of all pre-ART patients who are enrolled in HIV chronic care and expected to have periodic follow-up visits. On the other hand, our finding regarding the association between less advanced disease stage and pre-ART LTFU was consistent with a study conducted in southern Ethiopia that employed a similar operational definition and study settings [5]. Furthermore, we found that the risk of pre-ART LTFU increased progressively with increasing CD4 cell count, indicating a dose response relationship. This finding was supported by the study conducted in South Africa where the probability of returning to care dropped substantially for patients with higher CD4 cell count [10]. Part of the explanation for the increased odds of pre-ART LTFU among patients with better immune status or less advanced disease stage could result from patients’ self-assessment of feeling healthy and, therefore, not in need of health care [20].

The other contributory factor to pre-ART LTFU identified in this study was lack of documentation of patients’ next appointment date by clinicians. Studies conducted in Malawi and Kenya also identified patients’ charts that did not have the next appointment date specified, but excluded them from the analysis [14]. We included these individuals without recorded appointment date because lack of documentation itself might be indicative of the type of service patients received about specific follow-up visit days and could have an effect on patients’ follow-up. Indeed, this study found that patients without a record of the next visit date had a high chance of being LTFU during the pre-ART period. This could be because a clinician who failed to record the next appointment date on the patient’s chart might also omit to provide written or verbal information for the patient about the specific date of the next visit.

The main limitation of the study was the lack of or missing data regarding baseline socio-demographic and clinical variables and also our inability to locate some of the patient charts during the data collection period. Charts with no baseline information on the intake form, especially demographic data, and chart numbers that could not be found during random sampling were replaced by the next study subject (number) in the list of the study population. The rate of replacement of samples was low (5.5%). The other limitation was the study setting that was confined to only one university hospital, and hence the result might not be extrapolated to other locations.

## Conclusions

Our study found that patients who were less ill were more likely to be LTFU during the pre-ART period. The risk of pre-ART LTFU increased progressively with increasing CD4 cell count. These findings highlight the importance of giving greater attention to the care of pre-ART patients, starting from the time that they learn of their HIV positive serostatus. The standard of care for pre-ART patients needs to be revised to include measures to encourage follow-up, including early initiation of ART. Efforts to prevent pre-ART LTFU should be strengthened through mechanisms that proactively indentify and manage pre-ART patients who are likely to be non-adherent to care and become LTFU. There should be standardized definition of pre-treatment LTFU and appointment system that can monitor and easily flag-out missed appointment patients. Intensive adherence counseling and adequate information should be given to patients at-risk of LTFU at each visit especially during the first visit. The completeness of the medical records, the standard of record keeping and obstacles to retrieving charts also indicate a serious problem that needs due attention from clinicians and data personnel.

## Abbreviations

AIDS: Acquired Immune Deficiency Syndrome; ART: Anti-Retroviral Treatment; GUH: Gondar University Hospital; HIV: Human Immune-deficiency Virus; LTFU: Loss to Follow-up; PLHIV: People Living with HIV/AIDS; Pre-ART: Pre-Antiretroviral Treatment; WHO: World Health Organization.

## Competing interests

The authors declare that they have no competing interests.

## Authors’ contributions

IA had primary responsibility in the process of conceptualization and research design, data collection, data analysis and interpretation, and manuscript drafting and revision. STG and SL assisted in the conceptualization and design of the study, development of data collection instruments, interpretation of the results, and preparation of the manuscript. MD participated in the preparation of the manuscript and revising it critically for important intellectual content. All authors read and approved the final manuscript.

## Authors’ information

IA: RN, MPH; Care and Support Advisor at International Training and Education Center for Health-Ethiopia. He has expertise in HIV/AIDS treatment, care and support. STG: MPH; Research Scientist at University of Washington, Department of Global Health. She has expertise in Epidemiology and Bio-cultural Anthropology.

SL: MPH in Epidemiology; Researcher and Instructor at Addis Continental Institute of Public Health. MD: MD, MPH, PhD; Associate Professor at Addis Continental Institute of Public Health, Department of Public Health. She has expertise in Tuberculosis and HIV/AIDS Programs.

## Pre-publication history

The pre-publication history for this paper can be accessed here:

http://www.biomedcentral.com/1471-2458/13/867/prepub
